# Clinical and Laboratory Performance of ACTIVA BioACTIVE Restorative in Primary Teeth: A Systematic Review of Pediatric Evidence

**DOI:** 10.3390/jcm15010373

**Published:** 2026-01-04

**Authors:** Malina Popa, Stefania Dinu, Magda Mihaela Luca, Bogdan Andrei Bumbu, Edita Maghet, Romina Georgiana Bita

**Affiliations:** 1 Pediatric Dentistry Research Center (Pedo-Research), Department of Pediatric Dentistry, Faculty of Dental Medicine, “Victor Babes” University of Medicine and Pharmacy, Eftimie Murgu Square 2, 300041 Timisoara, Romania; popa.malina@umft.ro (M.P.); dinu.stefania@umft.ro (S.D.); 2 Department of Dental Medicine, Faculty of Medicine and Pharmacy, University of Oradea, 410073 Oradea, Romania; 3 Faculty of Dental Medicine, “Victor Babes” University of Medicine and Pharmacy, Eftimie Murgu Square 2, 300041 Timisoara, Romania; edita.maghet@umft.ro; 4 Department II, Radiology and Medical Imaging, General and Dento-Maxillary Imaging, Faculty of Dental Medicine, “Victor Babes” University of Medicine and Pharmacy, Eftimie Murgu Square 2, 300041 Timisoara, Romania; romina.bita@umft.ro

**Keywords:** dental caries, tooth, deciduous, composite resins, glass ionomer cements, calcium compounds

## Abstract

**Background and Objectives:** ACTIVA BioACTIVE Restorative is a resin–ionomer hybrid marketed as a bioactive material for pediatric restorations, yet its specific performance in primary teeth has not been systematically synthesized. The study aim was to evaluate clinical and laboratory outcomes of ACTIVA in primary dentition compared with established restorative materials. **Methods:** Following a PRISMA-aligned, OSF-registered protocol, PubMed, Scopus, and Web of Science were searched to 18 October 2025 for full-text clinical trials and in vitro studies involving ACTIVA in primary teeth or primary dentin. Eligible designs included randomized or prospective clinical studies and standardized in vitro experiments. Primary outcomes were clinical and radiographic success; secondary outcomes included placement time, shear bond strength, and calcium-ion release. **Results:** Three randomized clinical trials (86 children, 305 restorations) and one in vitro study met the inclusion criteria. At 12 months, clinical success with ACTIVA ranged from 97.5 to 97.8% versus 95.0–97.8% for bulk-fill and conventional composites. At 24 months, a split-mouth trial reported clinical success of 93.0% for ACTIVA and 95.3% for compomer, with radiographic success of 86.0% and 88.3%, respectively, remaining within the predefined non-inferiority margin. ACTIVA required a mean of 2.4 ± 0.6 min less placement time than compomer. In vitro, shear bond strength to primary dentin was higher for ACTIVA than for an RMGIC (4.29 ± 0.65 vs. 2.47 ± 0.32 MPa), with greater calcium-ion release at 21 days (0.77 ± 0.13 vs. 0.53 ± 0.12 ppm). **Conclusions:** Within 1–2 years of follow-up, ACTIVA shows clinical performance in primary molars comparable to compomer, bulk-fill, and conventional composites, while offering shorter placement time and favorable bio-interactive behavior.

## 1. Introduction

Dental caries in primary teeth remains one of the most common chronic conditions of childhood, with meta-analyses indicating that almost half of children worldwide have caries in their primary dentition and more than half in their permanent teeth [1]. Early-childhood caries (ECC) is particularly pervasive, affecting a large proportion of preschoolers and showing strong gradients by socioeconomic status, geographical region, and national income level [2]. Beyond the high prevalence, untreated carious lesions have measurable systemic and psychosocial consequences: children with severe caries often show impaired growth trajectories and lower body weight, with improvement after dental treatment [3], and multiple studies have linked untreated or advanced ECC with worse oral health–related quality of life (OHRQoL) for both children and caregivers [4]. In many settings, the burden is concentrated in socially disadvantaged groups, where access to regular care and behavior guidance is limited. Clinically, restoring carious primary molars is challenging: young children often have limited cooperation and short attention spans; achieving optimal isolation is difficult; and extensive cavitation frequently coincides with thin residual dentin and high caries activity. Current guidelines emphasize minimally invasive approaches and careful material selection, recognizing that not every lesion requires restoration and that all restorations have finite longevity [5]. In this context, restorative options must balance biological compatibility, technical simplicity, and durability under demanding behavioral and moisture conditions typical of pediatric practice.

The term “bioactive restorative” is generally applied to materials that go beyond passive gap sealing, instead exchanging ions with the surrounding environment and promoting mineral deposition at the tooth–material interface. Such materials typically release and, in some cases, recharge fluoride, calcium, and phosphate ions, with the aim of buffering pH fluctuations and supporting remineralization of adjacent tooth structure [6,7,8,9]. Conventional high-viscosity glass ionomer cements and resin-modified glass ionomers (RMGICs) are valued for chemical adhesion and fluoride release, but clinical trials in primary molars underscore limitations in wear resistance, marginal integrity, and fracture resistance under occlusal load [6]. Newer “bioactive” or “bio-interactive” formulations—such as modified RMGICs and hybrid materials—have been developed to improve mechanical performance while maintaining ion release [7,8,9]. ACTIVA BioACTIVE Restorative (Pulpdent) is marketed as an “ionic resin” combining a resin-composite matrix with a polyacid-modified, glass–ionomer–like phase. Laboratory and manufacturer data suggest that its reactive glass fillers release fluoride and calcium, while the resin phase provides light-curing and improved flexural properties. Time-dependent studies on related ionomer systems show that adhesion to enamel and dentin and sustained fluoride release can be influenced by substrate condition and material composition [10].

Most early clinical experience with ACTIVA was accumulated in permanent posterior teeth, where it was positioned as an alternative to conventional resin composites. Randomized controlled trials in adults and adolescents have reported comparable short- to medium-term performance between ACTIVA and nanohybrid or bulk-fill composites, with similar rates of marginal discoloration, postoperative sensitivity, and secondary caries over periods up to two years [7,8,9]. Network meta-analyses of bioactive restorative materials—encompassing glass-ionomer–based systems, giomers, and resin composites with bioactive components—generally suggest that some bioactive formulations may reduce secondary caries risk relative to conventional resin composites, although evidence is heterogeneous and often limited by short follow-up and small sample sizes [8,9]. Importantly, these syntheses focus predominantly on permanent dentition and aggregate multiple products under the “bioactive” label, making it difficult to isolate material-specific performance for ACTIVA. Anatomical and histological differences between primary and permanent teeth—including thinner, more permeable dentin and larger pulp chambers—as well as behavioral factors such as higher caries risk, dietary patterns, and oral-hygiene variability in young children, limit the generalizability of adult data to pediatric practice [5].

Evidence on ACTIVA BioACTIVE Restorative in primary teeth is emerging but remains limited. A small number of pediatric randomized clinical trials and laboratory studies have evaluated ACTIVA in primary molars or primary dentin, generally suggesting short-term clinical performance comparable to commonly used materials and measurable ion-release behavior under standardized conditions [11,12,13,14,15]. However, existing data are dispersed across heterogeneous study designs, follow-up durations, and outcome criteria, and no synthesis has focused specifically on primary dentition.

Bench studies provide additional insight into how ACTIVA interacts with primary dentin. In a controlled in vitro study, Bhatia et al. compared ACTIVA with Fuji II LC (RMGIC) using flattened occlusal dentin from extracted non-carious primary molars. ACTIVA exhibited significantly higher shear bond strength (4.29 ± 0.65 MPa) than Fuji II LC (2.47 ± 0.32 MPa) and released more calcium ions at all evaluation periods, reaching 0.76 ± 0.12 ppm at 21 days compared with 0.42 ± 0.07 ppm for Fuji II LC [14]. These findings suggest that, at least on primary dentin, ACTIVA can create a stronger micromechanical–chemical interface while maintaining a sustained ion-release profile. Other in vitro work has examined microleakage and interfacial behavior when ACTIVA is compared with Fuji II LC or modified RMGICs incorporating chitosan; such studies generally report lower microleakage scores for ACTIVA at the cervical margin, supporting the notion that its resin–ionomer hybrid structure may better compensate for polymerization shrinkage and thermal cycling [15]. Time-dependent adhesion and fluoride-release experiments on resin-modified glass ionomer systems more broadly underscore how substrate condition (sound vs. demineralized enamel/dentin) and material formulation influence both bond strength and the capacity to deliver beneficial ions over weeks to months [10].

Despite the growing body of pediatric data, no previous systematic review has focused exclusively on ACTIVA BioACTIVE Restorative in primary teeth. Existing syntheses either pool ACTIVA with diverse bioactive materials—such as conventional and resin-modified GICs, giomers, and calcium silicate–based restoratives—or consider ACTIVA primarily in permanent dentition [8,9]. Yet the clinical scenario where simplified, biologically compatible, and moisture-tolerant restorative materials may offer the greatest advantage is precisely the restoration of primary molars in young children, who often present with high caries risk, behavioral management challenges, and limited tolerance for multi-step adhesive procedures [1,2,5]. Furthermore, only a small number of in vitro studies have examined ACTIVA’s bonding and ion-release behavior specifically on primary dentin [14,15].

The present systematic review therefore aims to synthesize all available full-text evidence on ACTIVA BioACTIVE Restorative used in primary teeth, addressing two key questions: (i) How does ACTIVA perform clinically, compared with established pediatric materials (compomer, bulk-fill resin composite, conventional resin composite), in terms of survival, failure modes, and chairside efficiency? (ii) What is known about its bonding, ion-release, and interfacial behavior when applied to primary dentin and enamel? By collating study-level numerical outcomes and critically appraising methodological quality, this review seeks both to clarify ACTIVA’s role in pediatric restorative dentistry and to serve as a learning framework for conducting focused systematic reviews on emerging “bioactive” materials in children.

## 2. Materials and Methods

### 2.1. Protocol and Registration

For this systematic review, we followed the main elements of the PRISMA-2020 statement (Table S1), including pre-specification of objectives, eligibility criteria, outcomes, and data extraction domains [16]. The study protocol was registered with the Open Science Framework with the code osf.io/c5jtg. The core research question was framed using the PICO approach: Population—primary teeth (in vivo) or primary tooth dentin (in vitro) in children; Intervention—ACTIVA BioACTIVE Restorative used as a permanent direct restorative material or bonded to dentin; Comparators—other direct restorative materials or cements or, in the absence of a control, single-arm follow-up; Outcomes—clinical and radiographic success/failure, USPHS or FDI scores, placement time, and, for in vitro studies, shear bond strength and ion release; Study designs—randomized or non-randomized clinical trials, prospective cohorts, and laboratory experiments with extractable numeric outcomes.

We specified at the outset that only full-text articles accessible through PubMed-linked or publisher websites would be eligible, in line with your requirement that all included studies must be fully available to the reviewer. Trials known only from abstracts or where access was blocked were excluded, even if they appeared highly relevant, such as some earlier clinical evaluations of ACTIVA in primary dentition cited by included trials.

We also decided not to include narrative reviews, letters, or purely descriptive technique reports. We divided outcomes into primary (clinical success/failure at the longest follow-up) and secondary (esthetic/functional/biologic scores, placement time, ion release, bond strength).

### 2.2. Eligibility Criteria

Eligibility criteria were defined around the PICO elements and applied consistently at both the title/abstract and full-text stages [12,13,14,15]. For the population, we included studies that either restored primary teeth in vivo (children, typically aged 4–10 years) or used extracted human primary teeth as substrates in vitro. Studies exclusively involving permanent teeth or mixed dentitions without separately reported data for primary teeth were excluded from the main evidence tables, although some are discussed narratively as contextual background.

For intervention, only studies where ACTIVA BioACTIVE Restorative (Pulpdent) was used as a direct restorative material or as the test material in bond-strength/ion-release experiments were eligible. Work involving other “bioactive” restoratives (giomers, calcium-silicate composites) without ACTIVA was excluded.

With regard to comparators, we accepted any other commonly used pediatric restorative material (compomer, bulk-fill composite, conventional resin composite, RMGIC), as well as single-group clinical follow-up, where ACTIVA was the only material assessed. For outcomes, clinical studies needed to report at least one quantitative measure of restoration performance, such as clinical and/or radiographic success rate, FDI/USPHS scores, or detailed counts of failures. In vitro studies had to provide numeric data on shear bond strength and/or ion release in primary dentin. Qualitative reports without extractable numbers were excluded. Finally, for designs, randomized controlled trials, split-mouth trials, and prospective longitudinal studies were all eligible; cross-sectional observational reports and retrospective chart reviews were excluded due to limited follow-up and outcome control. In vitro designs based on standardized specimens and clearly described testing protocols were eligible.

We also required that the study be available as full text via the PubMed link, publisher site, or an open-access repository. This led to exclusion of some pediatric ACTIVA trials and in vitro studies where only an abstract or paywalled content could be accessed through the available tools, even though they were cited in other articles. Language was restricted to English. No limits on publication year were applied.

### 2.3. Information Sources and Search Strategy

We searched PubMed, Scopus, and Web of Science using combinations of Medical Subject Headings (MeSH) and free-text terms, from database inception to 18 October 2025. The core search string combined four concepts: (i) the material (“ACTIVA bioactive” OR “Activa BioACTIVE Restorative”), (ii) dentition (“primary teeth” OR “primary molars” OR “deciduous teeth” OR “pediatric”), (iii) restorative context (“class II” OR “restoration” OR “restorative” OR “filling”), and (iv) study type (“clinical trial” OR “randomized” OR “shear bond strength” OR “calcium ion release”). We complemented this with more general searches on “bioactive restorative primary teeth ACTIVA,” and we manually screened the reference lists of key articles. No filters on study design or publication year were initially applied to maximize sensitivity; during screening, we narrowed down to eligible designs.

All search results were imported into a spreadsheet, where duplicates (for example, the same BMC article appearing under PubMed and the publisher site) were removed manually. Titles and abstracts were first screened for mention of ACTIVA, primary teeth, or pediatric populations. Articles that clearly dealt only with permanent dentition or non-ACTIVA bioactive materials were excluded. Full-text access was then checked.

**Figure 1:**
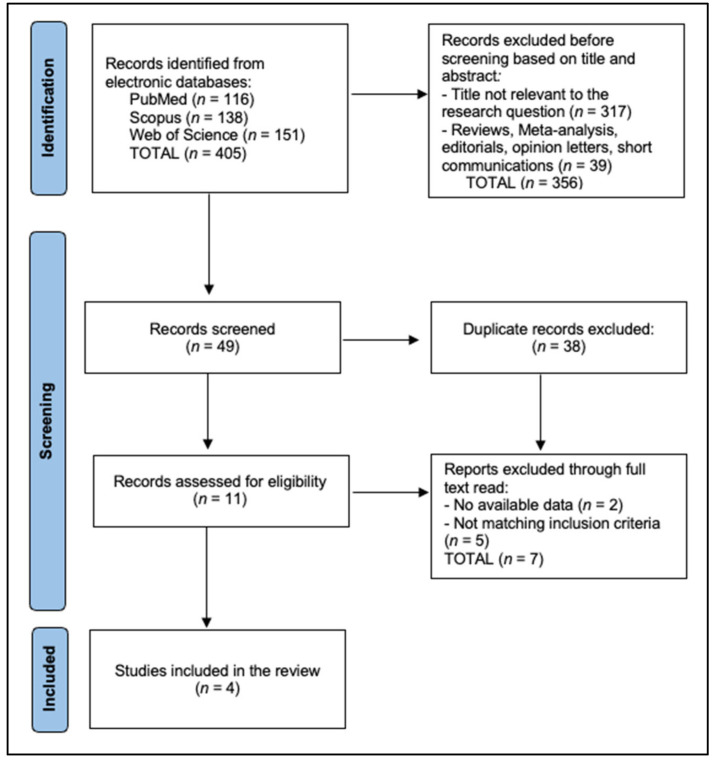
PRISMA flowchart diagram.

The PRISMA flowchart illustrates the selection process for studies included in the review. An initial search of electronic databases identified 405 records (PubMed *n* = 116, Scopus *n* = 138, Web of Science *n* = 151). After screening titles and abstracts, 356 records were excluded because they were not relevant to the research question (*n* = 317) or were reviews, meta-analyses, editorials, opinion letters, or short communications (*n* = 39), leaving 49 records to be screened. Of these, 38 were removed as duplicates, and 11 full-text articles were assessed for eligibility. Following full-text review, seven reports were excluded, either due to lack of available data (*n* = 2) or because they did not meet the inclusion criteria (*n* = 5). Ultimately, four studies fulfilled all criteria and were included in the final qualitative synthesis, as seen in Figure 1.

### 2.4. Risk of Bias Assessment and Data Synthesis

Risk of bias was assessed for the three included randomized clinical trials using the Cochrane RoB 2.0 framework (Table 1) [11,12,13]. Certainty of evidence (GRADE) was not formally applied in this review. The available body of evidence included only three small randomized clinical trials with heterogeneous outcome criteria (FDI vs. USPHS), different comparators, and limited follow-up (12–24 months), which precluded meaningful certainty ratings across outcomes. In addition, only one in vitro study contributed laboratory endpoints, and GRADE is primarily intended for patient-important clinical outcomes rather than mechanistic laboratory measures. For these reasons, we did not include a GRADE certainty-of-evidence table and instead present a structured narrative synthesis emphasizing study design limitations and outcome heterogeneity.

## 3. Results

Table 2 summarizes the design and sample characteristics of three pediatric clinical trials and one in vitro experiment evaluating ACTIVA BioACTIVE Restorative in primary teeth [11,12,13,14]. Lardani et al. [12] conducted a parallel-group randomized controlled trial (RCT) in an Italian university clinic, enrolling 45 children (mean age 6.15 ± 0.98 years, range 5–9) and placing 90 ACTIVA and 89 SDR bulk-fill composite restorations, with a mix of class I (34 vs. 33) and class II (56 vs. 56) cavities per material. Bañón et al. [11] performed a split-mouth non-inferiority RCT in a Belgian university hospital, randomizing 21 children (20 analyzed) aged 5–10 years to receive 48 class II proximal restorations in primary molars, of which 43 ACTIVA and 43 Dyract eXtra compomer restorations were evaluable at 24 months. Shihabi et al. [13] ran a single-center split-mouth RCT in a pediatric clinic (country not reported), including 20 children aged 6–9 years and restoring 40 class II cavities in primary molars (20 ACTIVA, 20 microhybrid resin composite). In contrast, Bhatia et al. [14] conducted an in vitro experimental study on 12 extracted primary molars, preparing 6 ACTIVA and 6 Fuji II LC specimens for shear bond strength testing on flattened dentin surfaces and additional disks for ion-release assessment, without reporting patient age because the work was ex vivo.

Table 3 presents the clinical and radiographic success of ACTIVA compared with conventional materials at final follow-up across the included studies [11,12,13,14]. In the 12-month trial by Lardani et al. [12], clinical success based on FDI biological criteria (scores 1–3) was 88/90 ACTIVA restorations (97.8%) with 2/90 failures (2.2%), almost identical to SDR bulk-fill composite with 87/89 clinically acceptable restorations (97.8%) and 2/89 failures (2.2%); radiographic outcomes were not reported. In the 24-month split-mouth RCT by Bañón et al. [11], ACTIVA achieved 40/43 (93.0%) clinical success and 37/43 (86.0%) radiographic success, while the compomer Dyract eXtra reached 41/43 (95.3%) clinical success and 38/43 (88.3%) radiographic success, indicating small absolute differences of 2–2.3 percentage points in favor of the comparator. Shihabi et al. [13] reported overall USPHS-based success at 12 months of 39/40 (97.5%) for ACTIVA versus 38/40 (95.0%) for the microhybrid composite, with 1/40 (2.5%) and 2/40 (5.0%) failures, respectively, and without radiographic data.

Table 4 collates key secondary outcomes, including esthetic and biologic scores, placement time, plaque control, postoperative sensitivity, shear bond strength, and calcium-ion release across the four studies [11,12,13,14]. In the 12-month clinical trial by Lardani et al. [12], 60/90 ACTIVA restorations (67%) versus 53/89 SDR restorations (60%) were rated “excellent/very good” for esthetics according to FDI, while biological scores were “excellent” in 87/90 (97%) ACTIVA and 86/89 (97%) SDR restorations, with only two failures per group. Bañón et al. [11] found that ACTIVA could be placed 2.37 ± 0.63 min faster than Dyract eXtra, a statistically significant difference (*p* < 0.001), and reported an overall reduction in plaque index from a median of 1.1 at baseline to 0.6 at 12 months for the combined sample, without material-specific differences. In Shihabi et al. [13], USPHS “Alpha” ratings for marginal adaptation at 12 months were achieved in 38/40 (95%) ACTIVA restorations and 37/40 (92.5%) composite restorations, with the remaining restorations rated “Bravo” and no secondary caries or postoperative sensitivity in either group (0/40 cases). The in vitro study by Bhatia et al. [14] showed a markedly higher shear bond strength of ACTIVA to primary dentin (4.29 ± 0.65 MPa) compared with Fuji II LC (2.47 ± 0.32 MPa), and higher calcium-ion release at 21 days (0.76 ± 0.12 ppm vs. 0.42 ± 0.07 ppm), supporting a more robust adhesive interface and sustained ion release for ACTIVA in primary dentin.

Table 5 provides a more granular view of performance metrics, differentiating esthetic, functional, biological, and radiographic outcomes, as well as interfacial properties such as bond strength and ion release, for ACTIVA versus comparators [11,12,13,14]. In the trial by Lardani et al. [12], 60/90 ACTIVA restorations (66.7%) and 53/89 SDR restorations (59.6%) received FDI esthetic score 1 (“excellent”) at 12 months, while functional excellence was recorded in 75/90 (83.3%) vs. 69/89 (77.5%) restorations and biological excellence in 87/90 (96.7%) vs. 86/89 (96.6%), with no secondary caries or pulpal complications in either group (0/90 and 0/89). In the 24-month split-mouth trial by Bañón et al. [11], ACTIVA showed 1/43 tooth (2.3%) with endodontic radiographic complications and 4/43 teeth (9.3%) with secondary caries, yielding 6/43 teeth (14.0%) with any radiographic complication, compared with 2/43 (4.7%), 3/43 (7.0%), and 5/43 (11.6%), respectively, for Dyract eXtra. Shihabi et al. [13] reported higher proportions of USPHS rating “A” at 12 months for ACTIVA compared with conventional composite across anatomic form (17/20, 85% vs. 9/20, 45%), marginal adaptation (17/20, 85% vs. 11/20, 55%), and marginal discoloration (20/20, 100% vs. 18/20, 90%), while secondary caries ratings “A” were identical (19/20, 95% in both groups); globally, ACTIVA restorations were classified as 85% success, 5% acceptable, and 10% failure, versus 45% success, 55% acceptable, and 5% failure for composite. Finally, Bhatia et al. [14] documented higher shear bond strength for ACTIVA (4.29 ± 0.65 MPa, median 3.90) compared with Fuji II LC (2.47 ± 0.32 MPa, median 2.34), predominantly adhesive failures in both groups, and consistently greater Ca^2+^ release by ACTIVA at 24 h (0.371 ± 0.053 ppm vs. 0.198 ± 0.074 ppm), 7 days (0.463 ± 0.017 vs. 0.281 ± 0.041 ppm), 14 days (0.491 ± 0.076 vs. 0.430 ± 0.179 ppm), and 21 days (0.768 ± 0.127 vs. 0.525 ± 0.121 ppm).

**Figure 2:**
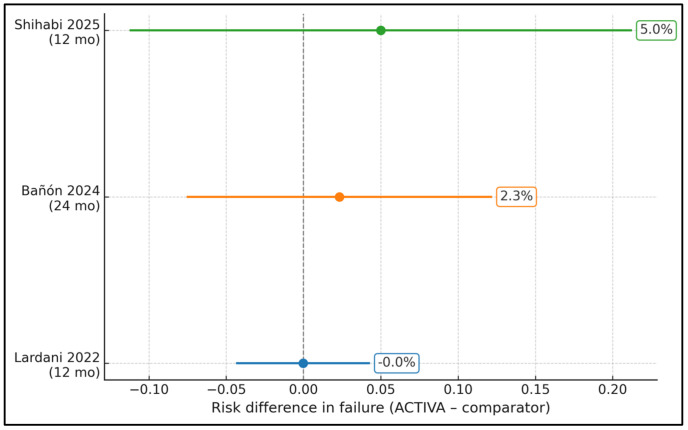
Risk difference in clinical failure (ACTIVA—comparator) by study [11,12,13].

Figure 2 shows the absolute risk difference in failure between ACTIVA and its comparator in each randomized trial, expressed as ACTIVA minus comparator. In Lardani’s study [12](12 months), failure was 2/90 (2.2%) for ACTIVA and 2/89 (2.3%) for SDR bulk-fill, giving a risk difference of essentially −0.0% (95% CI spans roughly −4.7% to +4.6%; blue line). In Bañón’s study [11] (24 months), failures were 3/43 (7.0%) for ACTIVA and 2/43 (4.7%) for Dyract eXtra, corresponding to a risk difference of +2.3% (orange line; 95% CI overlapping zero). In Shihabi’s study [13] (12 months), USPHS “failure” occurred in 2/20 (10%) ACTIVA restorations and 1/20 (5%) composite restorations, yielding a risk difference of +5.0% (green line; wide CI due to small *n*). All confidence intervals cross the vertical zero line, meaning no statistically significant difference in failure risk between ACTIVA and the various comparators over 1–2 years, even though point estimates slightly favor the comparator in the longer-term studies.

**Figure 3:**
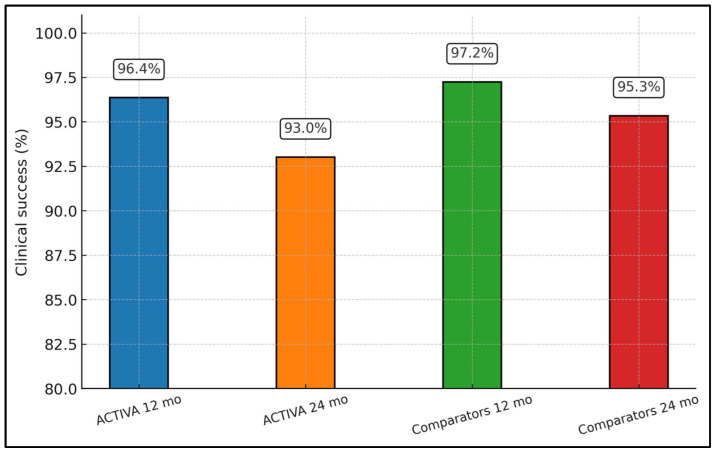
Subgroup analysis by follow-up duration (12 vs. 24 months).

At 12 months, ACTIVA and comparators are extremely close (96.4% vs. 97.2%), indicating virtually identical short-term performance. By 24 months, both groups show a small drop in success, but ACTIVA declines more (to 93.0%) than the compomer (95.3%), which is consistent with the non-inferiority (but not superiority) result of Bañón 2024. Overall, Figure 3 visually supports the message that ACTIVA is non-inferior in the first year and remains reasonably stable at two years, albeit with a slightly lower success rate than compomer over the longer interval.

## 4. Discussion

When the pediatric evidence is viewed in context, the available clinical trials indicate that ACTIVA achieves high 12–24-month success rates in primary molars and does not demonstrate statistically significant inferiority versus established comparators over the studied time horizons [11,12,13]. The single primary-dentin laboratory study identified higher shear bond strength and greater calcium-ion release for ACTIVA compared with an RMGIC under standardized conditions, supporting a plausible mechanistic basis for the early clinical findings [14]. Nevertheless, the small number of trials, modest sample sizes, and short follow-up mean that the observed equivalence should be interpreted as preliminary rather than definitive.

The in vitro component of our review, which demonstrated higher shear bond strength and greater calcium release for ACTIVA than Fuji II LC on primary dentin [14], is broadly consistent with other microleakage and bonding studies on ACTIVA and related “bioactive” formulations. Amaireh et al. tested class II restorations in primary molars and showed that when a conventional adhesive was used, ACTIVA exhibited microleakage at occlusal and gingival margins comparable to a microhybrid composite (Filtek Z250), while Vitremer displayed significantly greater leakage at the gingival margin [17,18,19,20]. Yao et al. compared ACTIVA and Fuji II LC with a new self-adhesive bulk-fill and adhesive/composite combinations; on flat dentin, immediate microtensile bond strength for ACTIVA was in the same range as conventional adhesive-mediated systems, but performance deteriorated on high C-factor cavity-bottom dentin, underlining the challenge of bonding in deep class I/II configurations [21]. François et al. likewise reported that ACTIVA’s shear bond strength to dentin and flexural properties were acceptable but not among the highest of recently developed self-adhesive bulk-fill materials, and that the use of a universal adhesive markedly influenced bonding performance [22].

The “bioactive” claims of ACTIVA are often interpreted in terms of ion release and remineralization, and our finding of sustained calcium release in primary dentin [14] is aligned with broader in vitro work on glass-ionomer–derived systems. However, recent mechanistic studies suggest that resin-based bioactive materials may not induce the same degree of true interface remineralization as calcium-silicate cements. Kunert et al. compared several resin-based “bioactive” materials—including ACTIVA BioACTIVE Base/Liner and ACTIVA Presto—with MTA, Biodentine, and other calcium-silicate cements, showing that the latter produced abundant surface precipitates that bridged the dentin–material interface, whereas ACTIVA-type materials generated only limited surface deposits and did not demonstrably remineralize the interface in SEM/EDX analysis [23]. Valverde-Rubio et al. further demonstrated that a bioactive resin (ACTIVA BioACTIVE Restorative) bonded well to sound dentin but showed poorer interaction and signs of interfacial separation in carious dentin compared with high-viscosity and resin-modified glass ionomers, which maintained more consistent chemical bonding across substrates [24]. These observations complement time-dependent adhesion/fluoride-release data for RMGICs [10] by suggesting that ACTIVA’s main biologic contribution in primary molars may be robust adhesion with modest ion release rather than strong interface remineralization, and that material selection should still consider dentin condition and caries activity.

When situating our pediatric findings within the broader literature on ACTIVA in permanent dentition, a consistent pattern of non-inferiority emerges. Bhadra et al. found no significant differences between ACTIVA and a nanohybrid composite in 1-year class II permanent tooth restorations, using modified USPHS criteria [19]. Other adult clinical data for bioactive or “ionic” resin-modified glass ionomer formulations show acceptable two-year performance, but without convincing superiority over conventional composites in terms of marginal integrity, discoloration, or secondary caries [7,8,9]. These adult studies are methodologically stronger and often larger than current pediatric trials, yet they are conducted in teeth with thicker dentin, different occlusal loading patterns, and lower caries risk than the high-risk pediatric populations considered here. Our review, together with the additional pediatric RCTs by Deepika and Bhavana [17,18], therefore supports the view that ACTIVA should be regarded as a clinically viable alternative rather than a clearly superior option: it appears to match resin composites and compomers in short-term performance, with potential advantages in handling and surface texture, but it does not yet demonstrate a consistently lower failure or secondary caries rate.

Finally, the accumulated evidence has several implications for pediatric restorative decision-making and future research. From a clinical perspective, ACTIVA’s ability to be bulk-placed, its relative tolerance to moisture, and its good short-term survival make it attractive for young or behaviorally challenging children, in line with guideline recommendations to prioritize simplified, biologically compatible techniques in high-caries-risk primary dentition [5]. At the same time, the slight trend toward better long-term performance of compomers or giomers in some domains [11,17], the limited evidence for true interface remineralization [23,24], and the technique sensitivity of ACTIVA at high-C-factor or carious dentin margins [20,21,22,24] argue against adopting it as a universal first-line material. ACTIVA can reasonably be integrated into a personalized restorative strategy for primary molars—particularly where reduced chair time and moisture tolerance are priorities—while recognizing that its clinical benefits appear to be equivalence and workflow facilitation rather than unequivocal biological superiority.

Regarding implications for clinical practice, the available evidence suggests that ACTIVA BioACTIVE Restorative is a clinically acceptable option for restoring class II cavities in primary molars, particularly in behaviorally challenging pediatric patients. One-year success rates above 97% and two-year success rates of 93.0% indicate that ACTIVA can provide survival comparable to compomers, bulk-fill, and conventional resin composites under routine clinical conditions. The documented reduction in placement time by approximately 2.4 min per restoration is clinically relevant in young or anxious children, where shorter procedures can improve cooperation and reduce the need for pharmacologic behavior management. In vitro data showing higher bond strength to primary dentin and greater calcium-ion release support its use in children with high caries risk or compromised tooth structure, where enhanced interfacial integrity and ion exchange may be advantageous. Taken together, these findings justify considering ACTIVA as a viable first-line material for primary molar restorations when a simplified, moisture-tolerant protocol is prioritized.

This systematic review is limited by the small number of available pediatric trials (three RCTs) and their modest sample sizes, which restrict the precision of effect estimates and preclude robust subgroup analyses. Follow-up periods were relatively short (12–24 months), so long-term performance and the durability of any bioactive effects remain uncertain. Clinical studies used different outcome criteria (FDI vs. USPHS) and sometimes reported composite endpoints, reducing comparability across trials and limiting the feasibility of formal meta-analysis. Risk of bias could not be fully excluded due to incomplete reporting of randomization procedures, blinding, and selective reporting. The in vitro data derive from a single laboratory study with standardized conditions that may not capture the complexity of the oral environment. Finally, only English-language, full-text articles accessible through major databases were included, potentially introducing language and availability bias.

## 5. Conclusions

This systematic review indicates that ACTIVA BioACTIVE Restorative achieves short-term clinical outcomes in primary molars that are broadly comparable to those of compomer, bulk-fill, and conventional resin composites, with 12-month success rates around 97% and acceptable performance up to 24 months. Its reduced placement time and favorable in vitro profile—higher bond strength to primary dentin and sustained calcium-ion release—support its use in pediatric patients, especially when moisture control and cooperation are suboptimal. However, current evidence does not demonstrate clear superiority over established materials, and data beyond two years are lacking. Larger, well-designed randomized trials with longer follow-up and standardized outcome reporting are needed to confirm the durability, caries-preventive potential, and cost-effectiveness of ACTIVA in primary dentition and to define its optimal indications within contemporary pediatric restorative protocols.

## Figures and Tables

**Table 1:** Risk of bias assessment for included randomized clinical trials (Cochrane RoB 2.0).

Study (Ref.)	Bias Arising from the Randomization Process	Bias Due to Deviations from Intended Interventions	Bias Due to Missing Outcome Data	Bias in Measurement of the Outcome	Bias in Selection of the Reported Result	Overall Risk of Bias
Lardani 2022 [12]	Low risk	Low risk	Low risk	Low risk	Some concerns	Some concerns
Bañón 2024 [11]	Low risk	Low risk	Low risk	Low risk	Low risk	Low risk
Shihabi 2025 [13]	Low risk	Low risk	Low risk	Low risk	Low risk	Low risk

RoB 2.0, revised Cochrane risk-of-bias tool for randomized trials; RCT, randomized clinical trial.

**Table 2:** Design and sample characteristics of included studies (primary teeth).

Study (Ref.)	Country/Setting	Design	Children (*n*)	Age (Years) Mean ± SD or Range	Restorations/Teeth (*n*, per Group)	Cavity Type (Class I/II)	Comparator Material(s)
Lardani 2022 [12]	Italy, university clinic	Parallel-group RCT, 12-month follow-up	45	6.15 ± 0.98 (range 5–9)	90 ACTIVA; 89 SDR bulk-fill composite	ACTIVA: 34 class I, 56 class II; SDR: 33 class I, 56 class II	SDR™ bulk-fill resin-based composite
Banon 2024 [11]	Belgium, Ghent University Hospital	Split-mouth non-inferiority RCT, 24-month follow-up	21 randomized; 20 analyzed	5–10 (mean NR)	48 teeth randomized; 43 ACTIVA and 43 Dyract at 24 months	All class II proximal cavities in primary molars	Dyract^®^ eXtra compomer
Shihabi 2025 [13]	Single-center pediatric clinic (country NR in excerpt)	Split-mouth RCT, 12-month follow-up	20	6–9 (mean NR)	20 ACTIVA and 20 resin-composite restorations (40 total)	Class II cavities in primary molars	Micro-hybrid resin composite (light-cured)
Bhatia 2022 [14]	India, university laboratory	In vitro experimental study	12 extracted teeth	NR (primary molars, ex vivo)	6 ACTIVA and 6 Fuji II LC specimens for SBS; separate disks for ion release	Not applicable (flattened dentin surfaces)	Fuji II LC resin-modified glass ionomer cement

Abbreviations: RCT, randomized controlled trial; SD, standard deviation; NR, not reported; SBS, shear bond strength; LC, light-cured.

**Table 3:** Clinical and radiographic success of ACTIVA vs. comparators at final follow-up.

Study (Ref.)	Follow-Up (Months)	Criteria Used	ACTIVA—Clinical Success *n*/N (%)	ACTIVA—Radiographic Success *n*/N (%)	Comparator—Clinical Success *n*/N (%)	Comparator—Radiographic Success *n*/N (%)
Lardani 2022 [12]	12	FDI (biological properties; failure defined as scores 4–5)	88/90 (97.8%) biologically acceptable (scores 1–3); 2/90 (2.2%) failures	NR	87/89 (97.8%) biologically acceptable; 2/89 (2.2%) failures	NR
Banon 2024 [11]	24	USPHS/Ryge (Alpha/Bravo = success; Charlie/Delta = failure)	40/43 (93.0%) clinical success; 37/43 (86.0%) radiographic success	37/43 (86.0%)	41/43 (95.3%) clinical success; 38/43 (88.3%) radiographic success	38/43 (88.3%)
Shihabi 2025 [13]	12	USPHS/Ryge (overall success; exact domains reported)	39/40 (97.5%) overall success; 1/40 (2.5%) failure	NR	38/40 (95.0%) overall success; 2/40 (5.0%) failures	NR
Bhatia 2022 [14]	Not applicable (in vitro)	Not applicable	NR	NR	NR	NR

Abbreviations: FDI, Fédération Dentaire Internationale criteria; USPHS, United States Public Health Service (Ryge) criteria; NR, not reported.

**Table 4:** Key secondary outcomes: esthetic/biologic scores, placement time, calcium-ion release, and shear bond strength.

Study (Ref.)	Outcome	ACTIVA (Mean ± SD or *n*/N, %)	Comparator (Mean ± SD or *n*/N, %)	Notes
Lardani 2022 [12]	Esthetic FDI score “excellent/very good” at 12 months	60/90 (67%)	53/89 (60%)	Esthetic properties slightly favored ACTIVA; differences not reported as statistically significant.
Lardani 2022 [12]	Biological FDI score “excellent” at 12 months	87/90 (97%)	86/89 (97%)	Both materials showed 2 failures (≈2.2%); one restoration per group rated “clinically sufficient”.
Banon 2024 [11]	Mean placement time difference (Dyract—ACTIVA)	2.37 ± 0.63 min faster for ACTIVA	—	ACTIVA took significantly less time to place than Dyract (*p* < 0.001).
Banon 2024 [11]	Plaque Index (PI), median (IQR): baseline vs. 12 months	PI baseline 1.1 (0.9); at 12 months 0.6 (0.275) (pooled across groups)	Same population	Oral hygiene improved over time; no between-material difference reported.
Shihabi 2025 [13]	USPHS “Alpha” scores (marginal adaptation) at 12 months	38/40 (95%) Alpha; 2/40 (5%) Bravo	37/40 (92.5%) Alpha; 3/40 (7.5%) Bravo	No significant differences; no secondary caries detected in either group.
Shihabi 2025 [13]	Post-operative sensitivity at 12 months	0/40 (0%)	0/40 (0%)	Both materials free of reported sensitivity.
Bhatia 2022 [14]	Shear bond strength to primary dentin (MPa)	4.29 ± 0.65	2.47 ± 0.32 (Fuji II LC)	Intergroup difference significant (*p* = 0.0122); dominant failure mode adhesive in both groups.
Bhatia 2022 [14]	Calcium-ion release at 21 days (ppm)	0.76 ± 0.12	0.42 ± 0.07 (Fuji II LC)	ACTIVA showed consistently higher Ca++ release at 1, 7, 14, and 21 days.

Abbreviations: FDI, Fédération Dentaire Internationale criteria; PI, plaque index; IQR, interquartile range; USPHS, United States Public Health Service (Ryge) criteria; MPa, megapascal; Ca^2+^, calcium ion; SD, standard deviation; NR, not reported.

**Table 5:** Performance metrics from included studies.

Study (Year)	Outcome Domain/Metric (Final Follow-Up)	ACTIVA BioACTIVE (or ACTIVA KIDS)	Comparator (Material)
Lardani 2022 [12]	Esthetic FDI—score 1 (“excellent”) at 12 mo	60/90 restorations (66.7%)	53/89 restorations (59.6%)—SDR
	Functional FDI—score 1 (“excellent”) at 12 mo	75/90 (83.3%)	69/89 (77.5%)
	Biological FDI—score 1 (“excellent”) at 12 mo	87/90 (96.7%)	86/89 (96.6%)
	Secondary caries/pulpal complications at 12 mo	0/90 (0%)	0/89 (0%)
Bañón 2024 [11]	Endodontic complications (radiographic) at 24 mo	1 tooth with peri-radicular radiolucency (1/43 = 2.3%)	2 teeth with peri-radicular radiolucency (2/43 = 4.7%)—Dyract eXtra
	Secondary caries (radiographic) at 24 mo	4/43 teeth (9.3%)	3/43 teeth (7.0%)
	Overall radiographic complications at 24 mo (peri-radicular + secondary caries + other)	6/43 teeth (14.0%)	5/43 teeth (11.6%)
	Mean restoration placement time (min)	NR (not numerically tabulated)	NR
Shihabi 2025 [13]	Anatomic form—USPHS rating “A” at 12 mo	85% of restorations (17/20)	45% of restorations (9/20)—conventional composite
	Marginal adaptation—rating “A” at 12 mo	85% (17/20)	55% (11/20)
	Marginal discoloration—rating “A” at 12 mo	100% (20/20)	90% (18/20)
	Secondary caries—rating “A” at 12 mo	95% (19/20)	95% (19/20)
	Success/acceptable/failure at 12 mo (global)	85% success, 5% acceptable, 10% failure	45% success, 55% acceptable, 5% failure
Bhatia 2022 [14]	Shear bond strength to primary dentin (MPa)	4.29 ± 0.65 (median 3.90); predominantly adhesive failures (83%)	2.47 ± 0.32 (median 2.34); adhesive 66.7%, cohesive 16.6%, mixed 16.6%—Fuji II LC
	Ca^2+^ release at 24 h (ppm, mean ± SD)	0.371 ± 0.053	0.198 ± 0.074
	Ca^2+^ release at 7 days (ppm)	0.463 ± 0.017	0.281 ± 0.041
	Ca^2+^ release at 14 days (ppm)	0.491 ± 0.076	0.430 ± 0.179
	Ca^2+^ release at 21 days (ppm)	0.768 ± 0.127	0.525 ± 0.121

Abbreviations: FDI, Fédération Dentaire Internationale criteria; mo, months; USPHS, United States Public Health Service (Ryge) criteria; MPa, megapascal; Ca^2+^, calcium ion; NR, not reported.

## Data Availability

The data presented in this study are available on request from the corresponding author.

## Supplementary Materials

The following supporting information can be downloaded at: https://www.mdpi.com/article/10.3390/jcm15010373/s1, Table S1: PRISMA 2020 Checklist.
